# Correlation Between Tumor Regression Grade and Clinicopathological Parameters in Patients With Squamous Cell Carcinoma of the Esophagus Who Received Neoadjuvant Chemoradiotherapy

**DOI:** 10.1097/MD.0000000000001407

**Published:** 2015-08-28

**Authors:** Yin-Kai Chao, Chun-Bi Chang, Wen-Yu Chuang, Yu-Wen Wen, Hsien-Kun Chang, Chen-Kan Tseng, Chi-Ju Yeh, Yun-Hen Liu

**Affiliations:** From the Division of Thoracic Surgery, Chang Gung Memorial Hospital, College of Medicine, Chang Gung University, Taoyuan, Taiwan (Y-KC, Y-JL); Department of Radiology, Chang Gung Memorial Hospital, College of Medicine, Chang Gung University, Taoyuan, Taiwan (C-BC); Department of Pathology, Chang Gung Memorial Hospital, College of Medicine, Chang Gung University, Taoyuan, Taiwan (W-YC, C-JY); Clinical Informatics and Medical Statistics Research Center, Chang Gung University, Taoyuan, Taiwan (Y-WW); Division of Hematology/Oncology, Chang Gung Memorial Hospital, College of Medicine, Chang Gung University, Taoyuan, Taiwan (H-KC); Department of Radiation Oncology, Chang Gung Memorial Hospital, College of Medicine, Chang Gung University, Taoyuan, Taiwan (C-KT).

## Abstract

The aim of this study was 2-fold: first, to assess the prognostic significance on overall survival (OS) of the 3-point tumor regression grade (TRG) in patients with esophageal squamous cell carcinoma (ESCC) who received neoadjuvant chemoradiotherapy (nCRT); second, to investigate the associations of TRG with the clinicopathological characteristics of the study patients.

A total of 357 ESCC patients were retrospectively enrolled. The 3-point TRG was determined by assessing the percentage of viable residual tumor cells (VRTC) in the resected specimens as follows: TRG 1, 0% VRTC; TRG 2, 1% to 50% VRTC; and TRG 3, >50% VRTC.

A TRG of 1, 2, and 3 was found in 32.2%, 38.9%, and 28.9% of the specimens, respectively. High TRG values were significantly associated with advanced pretreatment clinical stage, longer tumor length, and higher posttreatment tumor depth of invasion (yT), the presence of lymph node metastases (LNM), and lymphovascular invasion. We observed a stepwise decrease in 5-year OS rates with increasing TRG, as follows: 51% for patients with a TRG of 1, 28% for patients with a TRG of 2, and 22% for patients with a TRG of 3 (*P* < 0.001). TRG and LNM were independent predictors of OS in multivariate analysis. Notably, the prognostic impact of TRG on OS was greater in patients without LNM (*P* < 0.001) and ypT3 disease (*P* = 0.021).

TRG is independently associated with OS in ESCC patients treated with nCRT. The interrelationships between TRG, LNM, and depth of tumor invasion may improve the prognostic stratification in esophageal cancer.

## INTRODUCTION

Neoadjuvant chemoradiotherapy (nCRT) followed by surgery has become one of the standard treatments for locally advanced esophageal cancer.^1^ Accumulating evidence indicates that response to chemoradiation is the most important predictor of survival following nCRT.^2–4^ Unfortunately, the use of the TNM system for assessing tumor response following nCRT remains problematic.^5,6^ Although the concept of T or N downstaging has widely used to describe the response to nCRT, this approach has several significant shortcomings.^7–9^ For example, a minor regression of clinical T3 tumors may result in a downstage from cT3 to ypT2, whereas malignancies showing good response may continue to be staged as ypT3 in presence of persistent residual microscopic tumor foci in the adventitia (ie, no downstage). To circumvent this issue, a tumor regression grade (TRG) aiming at capturing the individual pattern of tumor regression after nCRT has been initially developed by Mandard and coworkers^10^ in a pilot study of 85 patients with esophageal carcinoma. The original study focused on the amount of treatment-induced fibrosis in relation to viable residual tumor cells (VRTC), resulting in a 5-point TRG as follows: no VRTC, rare VRTC, fibrosis outgrowing VRTC, VRTC outgrowing fibrosis, and absence of regressive changes. Unfortunately, the reproducibility and prognostic value of this approach has been challenged because of its complexity and lack of objective assessment criteria (which are mainly qualitative in nature).^11^ More recently, a quantitative estimation of TRG through the analysis of percentage VRTC with respect to the original primary tumor has been proposed.^6,12,13^ Specifically, a simplified 3-point TRG system using 50% VRTC as the cut-off point (ie, TRG 1: 0% VRTC; TRG 2: 1–50% VRTC; and TRG 3: >50% VRTC) has been shown to have a good interobserver reliability and may predict prognosis in patients with esophageal cancer.^14–17^ However, most published studies in the field were conducted in small sample sizes and mainly in patients with adenocarcinoma.^15–17^ Because the relationship between the 3-point TRG and prognosis in esophageal squamous cell carcinoma (ESCC) patients treated with nCRT remains unclear, we designed the present study with 2 main goals. First, we sought to assess the prognostic impact of the 3-point TRG in a large cohort of ESCC patients treated with nCRT. Second, we investigated the associations of TRG with the clinicopathological characteristics of the study patients.

## PATIENTS AND METHODS

### Patients

We retrospectively reviewed the records of 357 consecutive ESCC patients who underwent non-R2 surgical resection following nCRT at the Chang Gung Memorial Hospital (Taiwan) between January 1998 and October 2008. Cases who showed operative mortality were excluded because our main goal was to determine long-term outcomes rather than surgical deaths. We performed pretreatment staging using esophagram, chest and abdominal CT, and endoscopic ultrasound (EUS) with an ultrasonic miniprobe (UM2R/12 MHz or UM3R/20 MHz; Olympus, Inc., Tokyo, Japan). Patients were staged according to the 2010 (7th) AJCC staging criteria. The follow-up period continued until December 2014. The study protocol was approved by the Institutional Review Board of the Chang Gung Memorial Hospital. Informed consent was waived due to the retrospective nature of the study.

### Neoadjuvant Chemoradiotherapy and Surgical Resection

nCRT was based on 5-fluorouracil (1000 mg/m^2^ per day administered as a continuous infusion for 96 h on days 1–4 and 29–33) and cisplatin (75 mg/m^2^, given intravenously for 3 h on days 1–29). Patients received radiotherapy either sequentially to chemotherapy on days 8–29 (total dose = 30 Gy; 200 cGy/fraction) or concurrently with chemotherapy (total dose = 41.4 Gy; 180 cGy/fraction). Between 4 and 6 weeks after completion of nCRT, patients underwent a thorough restaging work-up based on esophagram, chest-to-abdomen CT, EUS, and bone scan. Elective esophagectomy was performed in the absence of contraindication. Patients were deemed eligible for surgery in presence of the following criteria: surgical fitness, with absence of heart failure (New York Heart Association class III or IV) and liver cirrhosis (>Child-Pugh class B), absence of a tracheoesophageal fistula, and absence of recurrent laryngeal nerve invasion. Patients with lesions of the middle/lower third of the esophagus were treated with a limited right thoracotomy or a thoracoscopic incision followed by reconstruction with an intrathoracic gastric tube (Ivor-Lewis procedure). Patients with neoplasms located in the upper third of the esophagus received the McKeown procedure (tri-incisional esophagectomy). All of the study patients underwent 2-field lymph node dissection. Pyloroplasty and jejunostomy tube feeding were performed when indicated. In order to assess the presence of anastomotic leakages, water-soluble contrast swallow studies were performed (when feasible) on postoperative day 10 or thereafter.

### Pathological Examination and Assessment of TRG

Surgical specimens were opened longitudinally and fixed in 10% formaldehyde overnight. In presence of residual tumors, representative sections were carefully examined for assessing the maximal depth of invasion and the relationships with the esophagus and stomach. In the absence of gross tumors, ulcerated or fibrotic areas were sampled and representative sections were submitted for examination.

A single experienced pathologist (CJ Yeh) carefully reviewed all of the original resected specimens from the 357 study patients. All slides were stained with hematoxylin and eosin. Independent of the nodal status, the extent of VRTC was determined in a semiquantitative fashion according to the estimated percentage of viable tumor cells in relation to the total cancer area. After examination of each specimen, the TRG was assigned and coded as follows: TRG 1: 0% VRTC; TRG 2: 1% to 50% VRTC; and TRG 3: >50% VRTC.

### Statistical Analysis

Categorical data are given as absolute frequencies and compared using the χ^2^ test or the Fisher exact tests (as appropriate). Continuous data are expressed as means and standard deviations and compared with the Student *t* test. Overall survival (OS)—calculated from the date of diagnosis to the date of death—was the main outcome measure. The Kaplan–Meier method was used to plot the survival curves, which were compared with the log-rank test. The main predictors of outcomes were investigated by means of univariate and multivariate analyses. Cox proportional-hazards models were fitted for multivariate analysis. After analyzing the interactions between the study variables, a backward stepwise procedure was to derive the best-fitting model. The SPSS statistical software (version 12; SPSS, Inc., Chicago, IL) was used for all calculations. The alpha error was set at 0.05 (2-tailed).

## RESULTS

### Associations Between TRG and the Clinicopathological Characteristics of the Study Patients

Of the 357 tumors examined, a TRG of 1, 2, and 3 was found in 32.2%, 38.9%, and 28.9% of the resected specimens, respectively. Overall, 3 pretreatment factors were found to be significantly related to TRG (Table 1). Higher TRG values (reflecting poor response following nCRT) were significantly associated with a more advanced pretreatment clinical stage, longer tumor length, and younger age. With regard to the association of TRG with pathological factors, we found that a higher TRG was significantly related to the risk of lymph node metastases (LNM). Specifically, pathologically positive lymph nodes were found in 8.7% of patients with a TRG of 1, 24.5% of patients with a TRG of 2, and 35.9% of patients with a TRG of 3, respectively (*P* < 0.001). The presence of advanced ypT3 tumors was more common and the rates of lymphovascular invasion were higher in patients with a TRG of 3 than in those with a TRG of 2 (*P* < 0.001).

**TABLE 1:**
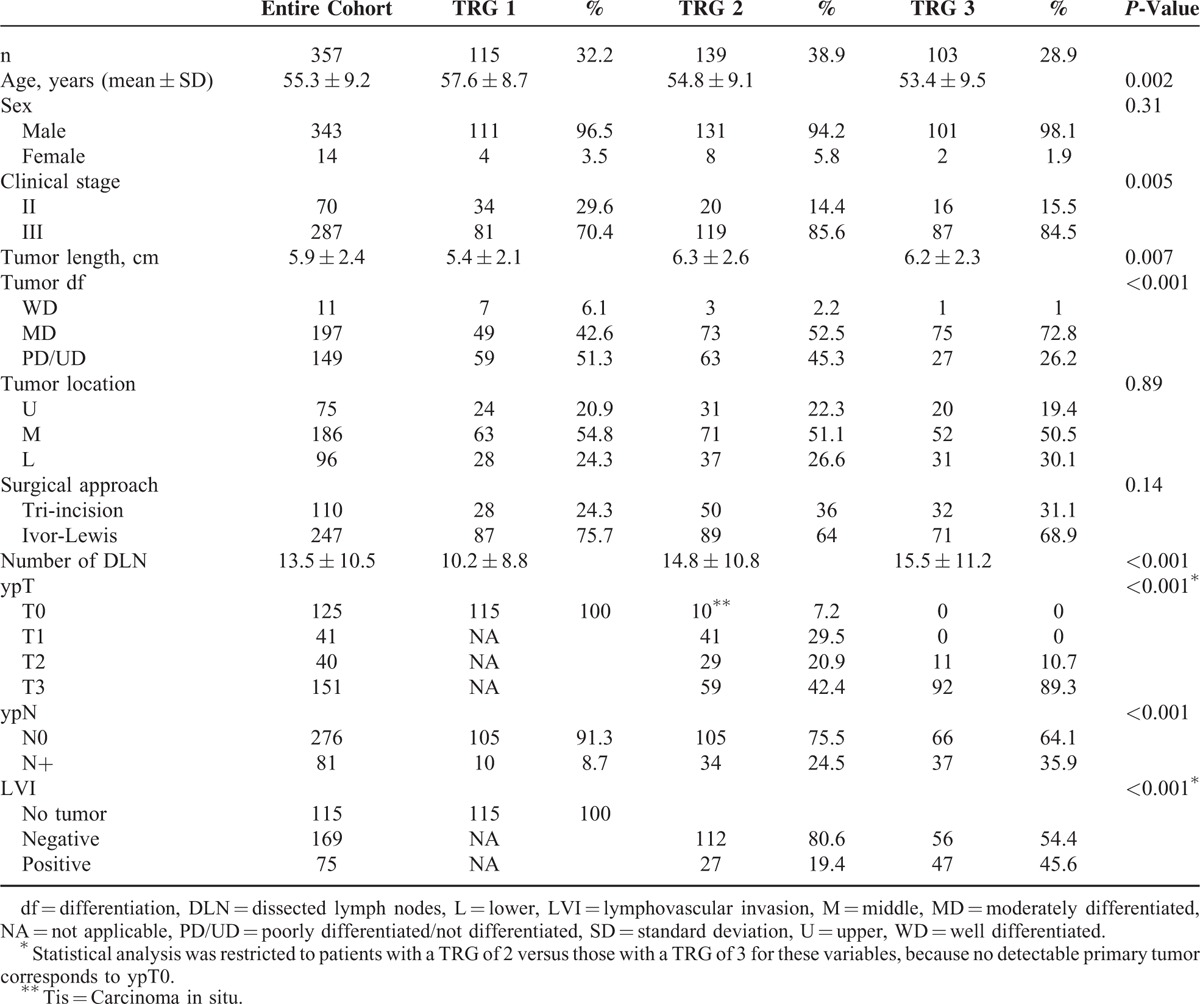
Associations Between the 3-Point Tumor Regression Grade (TRG) and Clinicopathological Factors

### TRG as a Prognostic Factor for Overall Survival

**FIGURE 1:**
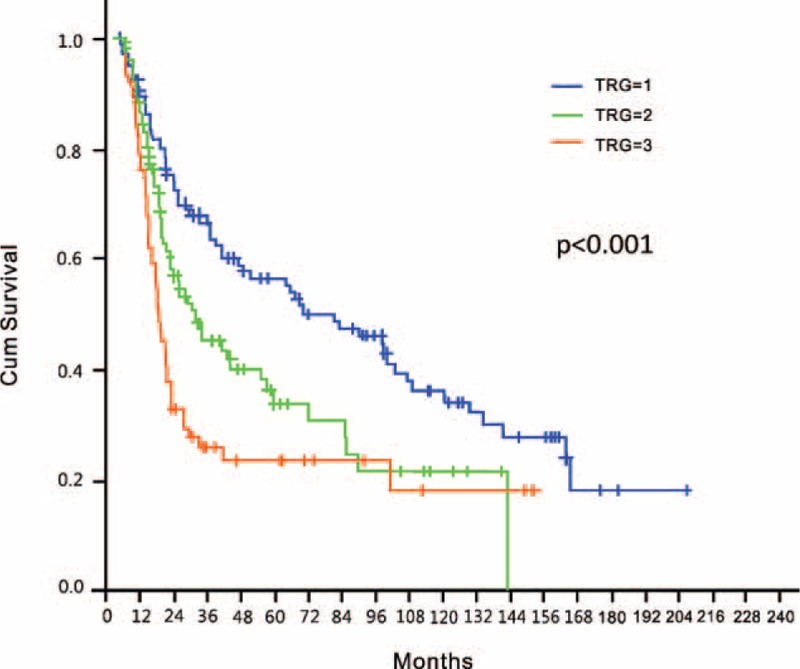
Overall survival in the entire study cohort according to the 3-point tumor regression grade (TRG).

In the entire study cohort, the 5-year OS after surgery with curative intent was 35%. We observed a stepwise decrease in 5-year OS rates with increasing TRG, as follows: 51% (median OS = 65 months) for patients with a TRG of 1, 28% (median OS = 25.4 months) for patients with a TRG of 2, and 22% (median OS = 16.8 months) for patients with a TRG of 3 (*P* < 0.001; Figure 1). Other factors significantly associated with OS in univariate analysis included ypT stage and the presence of LNM (Table 2). However, only TRG and LNM retained their independent prognostic significance in multivariate analysis after allowance for potential confounders (Table 2).

**TABLE 2:**
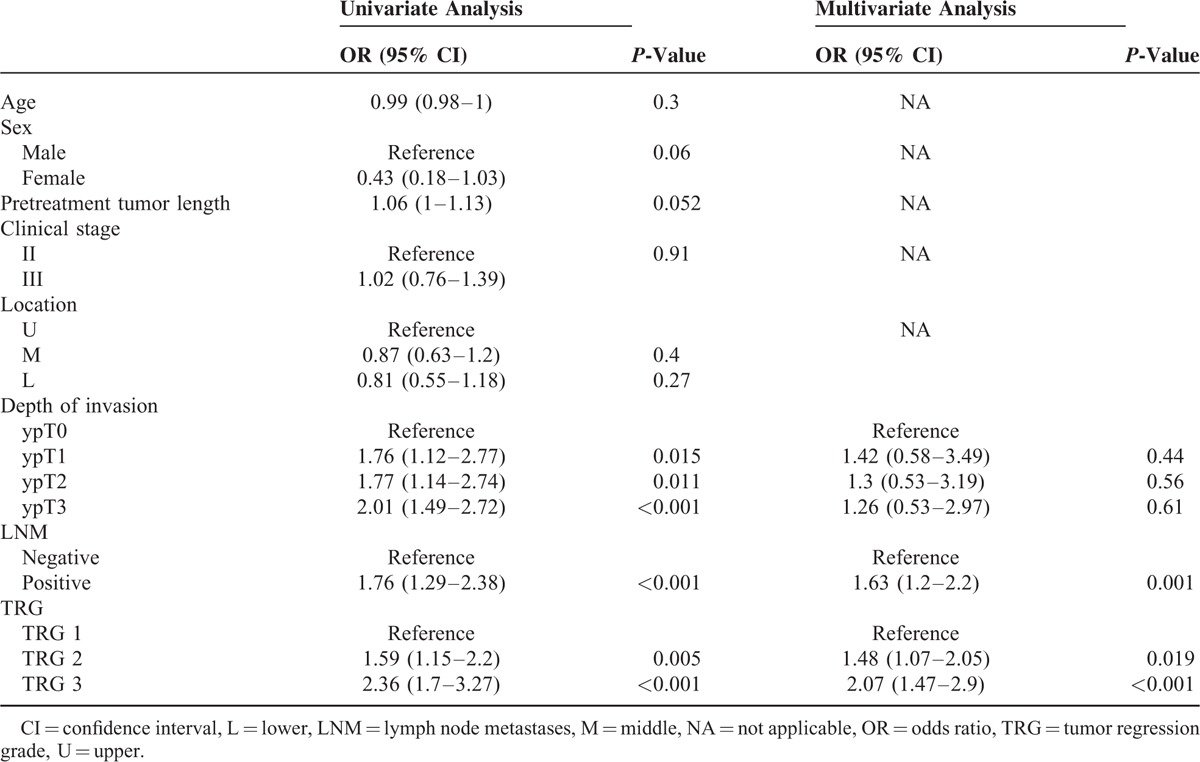
Univariate and Multivariate Analyses of Overall Survival

### Subgroup Analyses of the Prognostic Value of TRG in Relation to ypT Stage and the Presence of LNM

**FIGURE 2:**
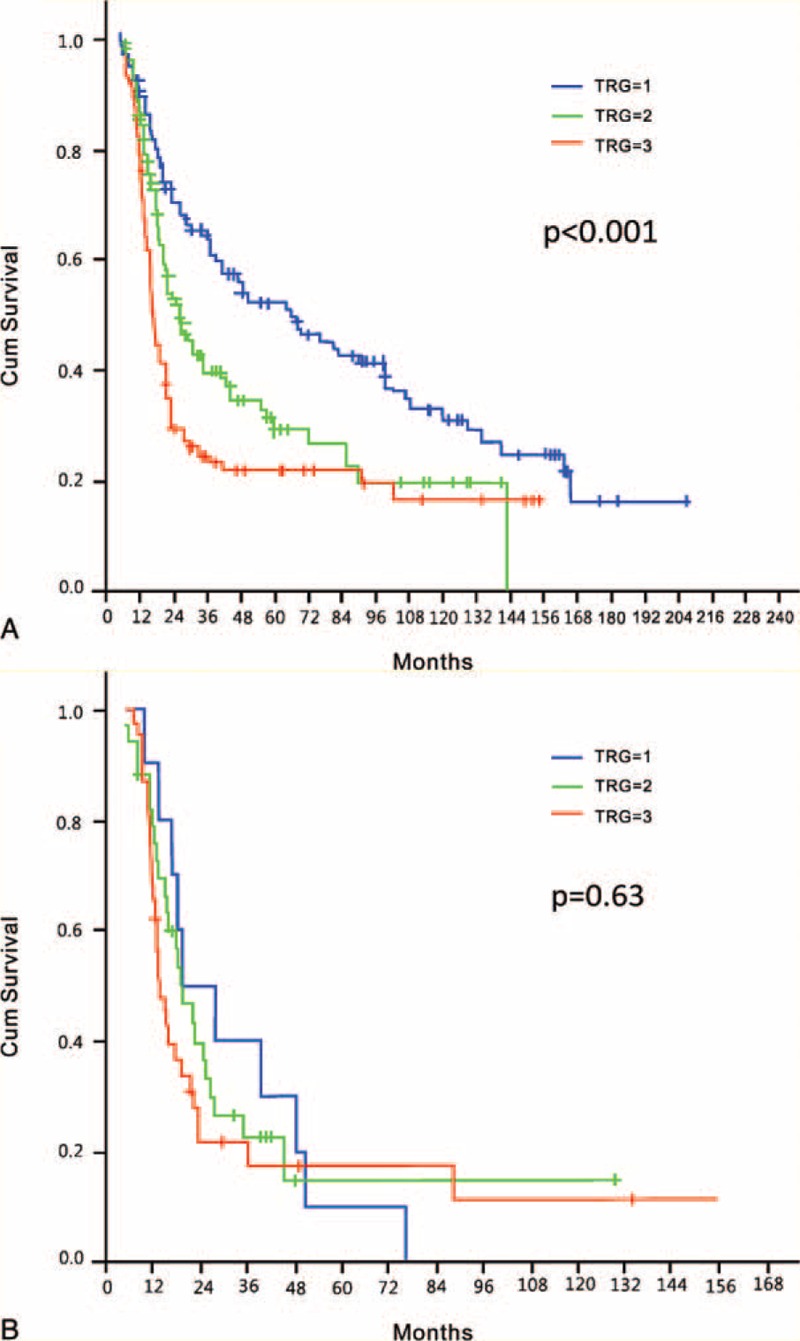
(A) Overall survival in patients without lymph node metastases according to the 3-point tumor regression grade (TRG). (B) Overall survival in patients with lymph node metastases according to the 3-point tumor regression grade (TRG).

**FIGURE 3:**
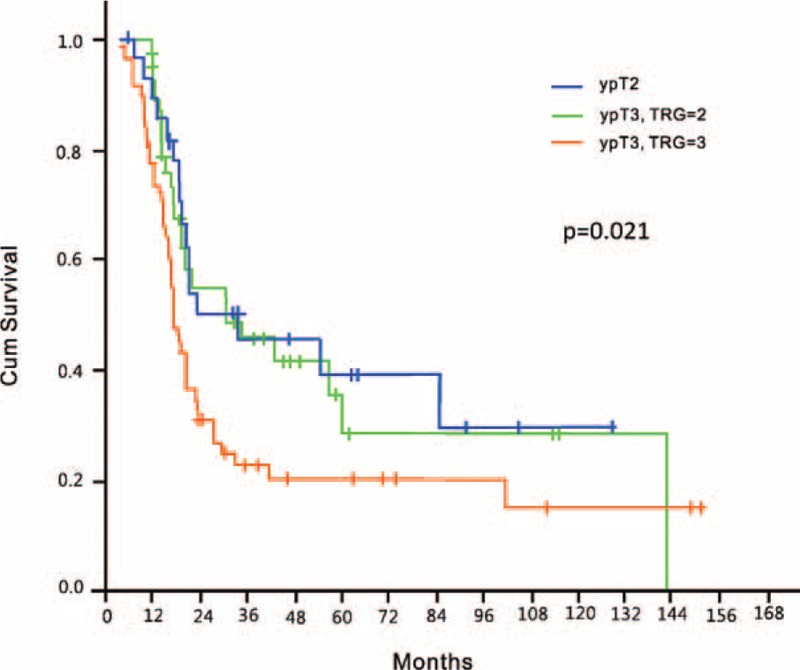
Overall survival in ypT2 and ypT3 patients (the latter stratified according to a tumor regression grade [TRG] of 2 vs. 3).

We finally performed subgroup analyses of the prognostic value of TRG in relation to ypT stage and the presence of LNM (Table 3). Because ypT0 corresponds to TRG 1, we restricted ypT subgroup analysis to ypT1–4a cases. We identified TRG as a significant prognostic factor for OS in patients without LNM (*P* < 0.001; Figure 2A) but not in presence of LNM (Figure 2B). We also found that TRG was significantly associated with OS in patients with advanced ypT3 stage (*P* = 0.02; Figure 3) but not in those with nonadvanced T categories (T1/T2).

**TABLE 3:**
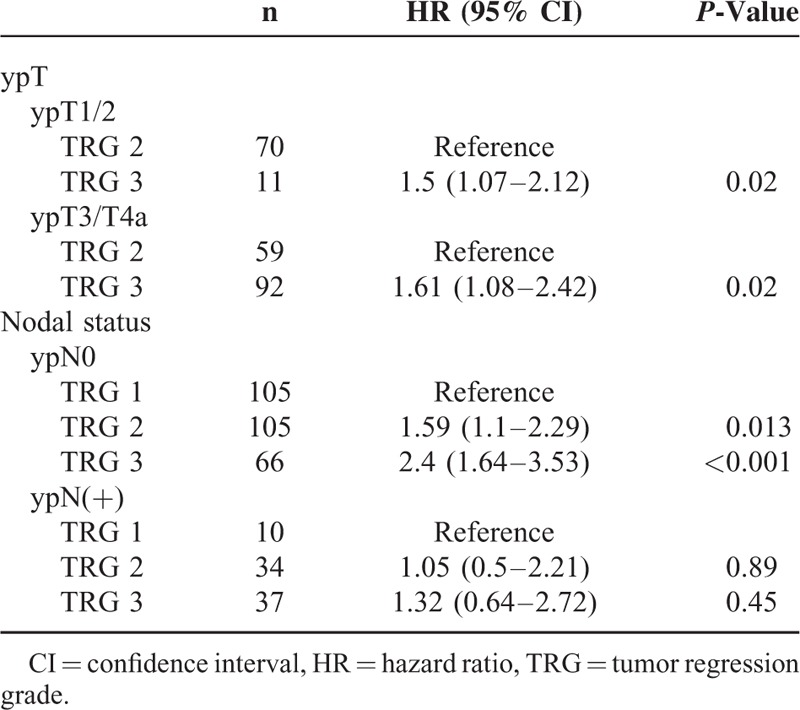
Subgroup Analyses According to the ypT and ypN Categories

## DISCUSSION

To our knowledge, this is the largest study to date to investigate the prognostic significance of the simplified 3-point TRG in a homogenous cohort of 357 ESCC patients (Table 4). After a careful pathological review of all resected specimens, we identified the extent of tumor regression following nCRT as an independent prognostic factor for OS. Notably, we also demonstrated that the prognostic impact of TRG on OS was modified by the T and N status. Specifically, TRG was a significant prognostic factor in patients without LNM and advanced ypT3 stage, whereas it did not show a statistically significant association with OS in patients with LNM and in those with nonadvanced T categories. Taken together, our findings indicate that the interrelationships between TRG, lymph node metastases, and depth of tumor invasion may improve the prognostic stratification in esophageal cancer.

**TABLE 4:**
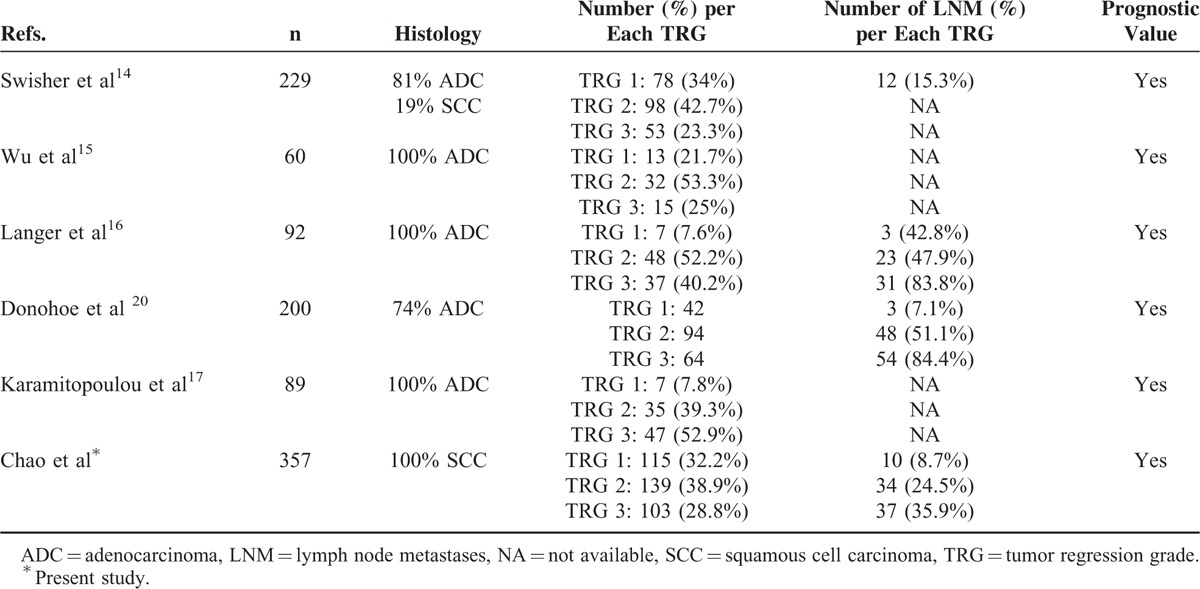
Published Studies Focusing on the Prognostic Significance of the 3-Point Tumor Regression Grade in Patients With Esophageal Cancer

The traditional radiobiological paradigm states that tumor eradication by any given amount of radiation depends on both tumor size and the extent of oxygenation.^18^ Herein, we demonstrated that malignancies with a shorter length and earlier clinical stages were more likely to achieve a better response following nCRT (Table 1), indirectly supporting the aforementioned hypothesis. Because the size of metastatic nodes is generally smaller than that of the primary tumor, the eradication of LNM is expected to be easier as compared with the primary malignancy after the administration of an equal amount of radiation. Although a favorable response to nCRT at the primary site was paralleled by a better clearance of metastatic nodes (Table 2), we nonetheless demonstrated that 8.5% and 24.5% of patients with a TRG of 1 and 2, respectively, had LNM. We believe that the persistence of LNM should be considered to reflect chemo- and/or radioresistance even in presence of a favorable response (low TRG) at the primary tumor site. Consequently, it is not surprising that TRG does not predict OS in this subgroup. Notably, the survival figures of these patients were poor regardless of their TRG (Figure 2B).

The spread of esophageal cancer generally follows a sequential pattern of invasion, starting from the innermost mucosa and submucosa, through the muscular layer, and final progression to the adventitia. In patients who do not undergo preoperative therapy, the depth of tumor invasion (defined by the T stage) is the most clinically relevant marker of local disease severity.^19^ However, this parameter has less prognostic significance in subjects who receive nCRT. It should be noted that tumor response to nCRT is anatomically unpredictable and does not necessary follow a reverse pattern compared with cancer invasion (ie, from the outer to the inner layer). A tumor classified as ypT3 may vary from a small residual cancer located in the adventitia (with complete clearance of tumor cells in the muscle layer as well as in the mucosa/submucosa) to a complete absence of regression accompanied by transmural invasion. In this scenario, the incorporation of TRG into ypT staging may offer superior prognostic stratification, especially in patients with advanced ypT3/4a disease (Table 3). Notably, the survival of patients with ypT3 (TRG 2) was significantly better than those with ypT3 (TRG 3) and similar to that observed in ypT2 cases (Figure 3). Interestingly, TRG did not show a similar prognostic impact in the ypT1/T2 subgroup. However, this lack of predictive value in the nonadvanced T1/T2 subgroup should be interpreted with caution. Because most of the ypT1/T2 cases had a TRG of 2, the small number of patients with a TRG of 3 might limit the reliability of the conclusions. Larger sample sizes are needed to shed more light on this issue.

Three main caveats of the present study merit comment. First, our conclusions should be interpreted with caution because of the retrospective study design and the long enrollment (1998–2008). Second, the radiation dose used for nCRT in the early study period (before 2007) was lower than that currently recommended (30 Gy vs. 40–45 Gy), potentially influencing the tumor regression patterns. Third, we cannot exclude an operator bias because TRG scores were assigned by a single pathologist and were not independently confirmed. Notwithstanding these limitations, the results of the present study may pave the way for an improved risk stratification of ESCC patients undergoing nCRT. This is especially important because of the growing use of induction therapies.

In conclusion, TRG is an independent predictor of OS in ESCC patients treated with nCRT. The interrelationships between TRG, lymph node metastases, and depth of tumor invasion may improve the prognostic stratification in esophageal cancer.
